# Analysis of Factors Influencing Spatial Distribution of Soil Erosion under Diverse Subwatershed Based on Geospatial Perspective: A Case Study at Citarum Watershed, West Java, Indonesia

**DOI:** 10.1155/2024/7251691

**Published:** 2024-01-11

**Authors:** Irmadi Nahib, Yudi Wahyudin, Fahmi Amhar, Wiwin Ambarwulan, Nunung Puji Nugroho, Bono Pranoto, Destika Cahyana, Fadhlullah Ramadhani, Nawa Suwedi, Mulyanto Darmawan, Turmudi Turmudi, Jaka Suryanta, Vicca Karolinoerita

**Affiliations:** ^1^Research Center for Geospatial, National Research and Innovation Agency of Indonesia (BRIN), Jalan Raya Jakarta Bogor KM 47 Cibinong, Bogor, West Java 16911, Indonesia; ^2^Faculty of Agriculture, Universitas Djuanda, Jl. Tol Ciawi No. 1, Ciawi, Bogor, West Java 16720, Indonesia; ^3^Research Center for Ecology and Ethnobiology, National Research and Innovation Agency, Jalan Raya Jakarta-Bogor Km. 46, Cibinong, Bogor, West Java 16911, Indonesia; ^4^Research Center for Food Crops, National Research and Innovation Agency, Jalan Raya Jakarta-Bogor Km. 46, Cibinong, Bogor, West Java 16911, Indonesia

## Abstract

The application of remote sensing data has been significant in modeling soil erosion. However, previous studies have fallen short in elucidating and lacked an understanding of the multifactor influencing erosion. This study addresses these limitations by employing the InVEST and the Geodetector models. Specifically, it aims (1) to delineate both spatial and temporal variations in soil erosion within the Citarum watershed from 2010 to 2020, (2) to identify the key drivers of soil erosion and unravel the underlying mechanisms, and (3) to identify the high-risk zones for soil erosion. Both models consider a range of natural predictors, including topography (slope factor), climate (precipitation factor), and vegetation cover (vegetation factor). In addition, they incorporate social parameters such as income per capita and population density, which interact with the watershed's position in the downstream, middle, and upper streams. The results reveal that, over a decade, the average soil erosion increased by 15.50 × 10^6^ tons, marking a 16.65% surge. The impact of factors varies significantly across different subwatershed areas. For example, fraction vegetation cover interactions influence upper- and middle-stream regions, while the downstream area is notably affected by precipitation interactions. The high-risk erosion areas in the watershed are primarily influenced by slope, precipitation, and fractional vegetation cover. In these areas, factors causing high erosion risks include slope, precipitation, and other environmental variables categorized into strata. The study highlights the varying influential factors in different watershed areas.

## 1. Introduction

Soil, as the interface of the biosphere, atmosphere, hydrosphere, and lithosphere, plays an essential role in maintaining life on Earth and the food system by providing essential resources for human beings and ecological systems [1]. Being a thin layer of Earth's skin, soil encounters various difficulties and dangers [2]. The depletion of soil resources is a significant global concern, with soil loss through erosion identified as a primary contributing factor [1]. Water-induced soil erosion significantly contributes to global soil degradation, depletion, and deterioration. In particular, tropical regions with high precipitation intensity have a higher possibility of soil erosion than other areas [3].

According to recent scientific statistics, the global average annual soil loss varies from 12 to 15 tons·ha^−1^·yr^−1^, posing a danger to both the natural environment and human existence [4]. Recent research by [5] indicates that the average rate of soil erosion in 2020 was 16.6 tons·ha^−1^·yr^−1^, ranging from 7.4 to 39.8 tons·ha^−1^·yr^−1^. Soil erosion compromises water quality, agricultural yield, and soil fertility and increases the risk of geological disasters, including landslides and debris flow [6]. In Indonesia, soil erosion rates range from 35 to 220 tons·ha^−1^ yr^−1^ [7], although there is no consensus on the exact amount. Some publications suggest that soil erosion in Indonesia can reach levels from 97.5 to 423.6 tons·ha^−1^·yr^−1^, but data for the 2020s are not yet available.

To design successful strategies for reducing soil erosion and restoring ecological balance, it is crucial to understand the causes and effects of soil erosion [8]. In 2015, the United Nations proposed a series of Sustainable Development Goals (SDGs), one of which is to “halt and reverse land degradation” [9]. Soil erosion is caused by multifaceted factors resulting from natural and anthropogenic activities, which often interact with one another [10]. Natural factors that have been shown to impact soil erosion significantly include changes in vegetation cover [11], land use types [12], soil types [13], rainfall, and slope [14]. In addition, anthropogenic factors resulting from human activities exacerbate soil erosion [15] as they lead to the overexploitation of ecosystem components such as soil and landscapes.

Soil erosion caused by rainfall and surface water flow is typically influenced by five key factors: (1) rainfall erosivity, (2) soil erodibility, (3) topography, (4) surface coverage, and (5) support practices. Soil erosion rates are lower in humid regions with existing forests and paddy fields than in areas with deforested lands, urban development, and steep upland agriculture [16]. Moreover, climate change expedites the rates of soil erosion through diverse mechanisms, such as changes in precipitation and temperature patterns, runoff, soil moisture, infiltration rates, biomass production, and alterations in land use [17].

Soil erosion has been a subject of human interest for centuries [18]. While direct field measurements of soil erosion can yield precise data on runoff and net soil loss, they are both time consuming and expensive when applied to estimate large-scale soil erosion [19]. Among various models of soil erosion that have been developed, the Universal Soil Loss Equation (USLE) [20] and the Revised Universal Soil Loss Equation (RUSLE) [21] are the most commonly utilized ones.

The RUSLE model has witnessed recent advancements. It has incorporated the concept of the sediment delivery ratio (SDR), which quantifies the proportion of sediment transported in a specific watershed section concerning the total erosion within that watershed [22]. This addition, coupled with runoff and sediment transport considerations, enables the RUSLE model to estimate sediment export and retention.

However, it is important to notice that calculating and creating models for soil erosion and understanding the impact of multiple factors and their interactions are difficult and complex tasks [23, 24]. Moreover, most of the research on soil erosion has been conducted in specific regions, with little comparison made between different geological settings. In addition, prior research efforts have often been inadequate in comprehensively explaining the various factors influencing soil erosion and frequently lacked a clear understanding of the driving forces behind the varying levels of soil erosion intensity. In conclusion, there are several issues with the current state of information on the driving forces behind soil erosion, including the need for more attention to the relationship among diverse elements and an unclear understanding of how these forces change as erosion intensity increases. This makes managing subtle soil erosion more challenging and, in some cases, hinders the sustainable use of land resources [25].

As stated by [26], spatial analysis approaches have the advantage of depicting the spatial distribution of soil erosion by identifying areas with high or low erosion intensity, e.g., the InVEST-SDR (Integrated Valuation of Ecosystem Services and Tradeoffs-Sediment Delivery Ratio) model [27]. It combines elements of the RUSLE with insights from studies by [28, 29]. By doing so, it computes the spatial distribution of soil erosion and sediment production in the watershed by calculating the ratio between soil erosion and sediment transport [30]. Nevertheless, relying solely on spatial analysis approaches, such as the InVEST-SDR model, is insufficient for directly quantifying the impact of factors driving soil erosion

Several studies used the Geodetector model to quantify the impact and the interaction of driving factors on the subject being studied [31–33]. The Geodetector model was proposed by Wang and Xu [34] at the Institute of Geographic Sciences and Natural Resources Research, Chinese Academy of Sciences, in 2015 [34]. It is a statistical method to detect spatial-stratified heterogeneity and identify the main driving forces of variables. Furthermore, it describes spatial variation similarities in geographic data by identifying spatial heterogeneity in geographical phenomena. It compares their spatial distributions by determining the impact of two independent variables on a dependent variable. The spatial distribution of two independent variables must be similar if they significantly impact a dependent variable [31, 35].

Integrating InVEST-SDR with the Geodetector model makes the approach more robust and comprehensive. It becomes possible to quantify soil erosion and understand the spatial patterns and drivers of erosion risk. This combined approach enhances decision-making by offering a more holistic view of how driving factors (land use, land cover, and environment) interact to impact soil erosion, ultimately leading to more effective soil erosion control and management strategies [36].

The Citarum watershed is one of Indonesia's national priority watersheds, serving as a vital water source for Bandung and Jakarta while reducing pollution and damage. Understanding hydrological conditions, urban and industrial expansion, economic growth, agriculture, fisheries, and hydroelectricity in West Java Province and Jakarta is crucial for maintaining the ecological services provided by the Citarum watershed [37]. Presidential Regulation Number 15 of 2018 introduced the Citarum Harum Program, aimed at the rehabilitation of the Citarum watershed. This nationwide program focuses on preventing damage and pollution while rehabilitating watersheds. The program assumes a vital role in tackling the notable issue of land degradation attributed to erosion in the Citarum watershed, primarily stemming from alterations in vegetation cover.

This research aims to improve the administration of regional ecosystem services and ecological management by identifying the causative agents of soil erosion under diverse subwatersheds in the Citarum watershed. The research comprises three specific goals. Initially, it aims to outline the spatial and temporal fluctuations in soil erosion within the Citarum watershed from 2010 to 2020. Second, the study seeks to utilize the Geodetector software to calculate the causal components of soil erosion and investigate their interconnectedness. Finally, the objective is to identify the high-risk zones for soil erosion.

The anticipated outcomes of this investigation will offer valuable perspectives on the fundamental factors contributing to soil erosion within the Citarum watershed. Furthermore, these findings will facilitate the formulation of practical approaches for managing and alleviating this issue. The utilization of the model enables the prioritization of interventions, identification of high-risk zones, and evaluation of the efficacy of various management strategies.

## 2. Materials and Methods

### 2.1. Study Area

The study was conducted in the Citarum watershed area, a 690,916 ha region located over eight regencies in West Java Province. Geographically, the Citarum watershed is situated between 106°51'36” and 107°51' E longitudes and 7°19' and 6°24' S latitudes. The climate of the region is characterized by a three-month period of precipitation totaling less than 40 mm and an average annual rainfall of 2,358 mm. The Citarum River mentioned in this context, utilized for electricity, agriculture, and freshwater supplies for the residents of numerous West Java regencies, is located inside the watershed and is spanned by three major dams: Saguling, Cirata, and Jatiluhur.

**Figure 1:**
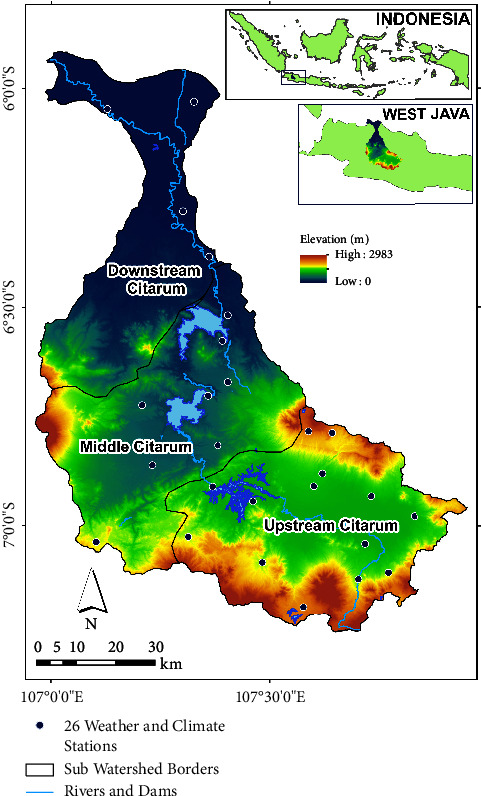
Map of the Citarum watershed, West Java, Indonesia.

The morphology of the area exhibits considerable diversity, encompassing hills and volcanic structures, featuring slopes that range from 5 to 15% at the base, 15 to 30% on the mountain slopes, and 30 to 90% at the mountain tops. The mountains upstream of the Citarum tributary are located at altitudes between 750 and 2,300 m above sea level (masl). The plains upstream, however, show volcanic structures with mild relief features [38, 39]. The location and the topography of the Citarum watershed are presented in Figure 1.

According to the landforms or morphological classification, the Citarum watershed can be classified into three divisions: upstream, middle stream, and downstream. In the upstream sections, (1) the topography of the upper section of the watershed takes the shape of a substantial basin referred to as the Bandung Basin, situated at elevations ranging from 625 to 2,600 meters above sea level. (2) The geological composition in the upper Citarum areas primarily consists of tuff, lava, breccia, and lapilli. (3) The average minimum temperature in the upper region of highland/mountainous areas is recorded at 15.3°C, and the average annual rainfall is 4,000 mm. (4) The soil type of the upper Citarum watershed consists of latosol (35.7%), andosol (30.76%), alluvial (24.75%), red yellow podzolic (7.72%), and regosol (0.86%) [40].

In the middle stream section, (1) the topography of the middle segment exhibits diverse morphological landforms, including plains (elevations between 250 and 400 meters above sea level), undulating hills (elevations between 200 and 800 meters above sea level), steep slope hills (elevations between 1,400 and 2,400 meters above sea level), and volcanic edifices. (2) Geological composition in the middle section encompasses volcanic sediments, ancient lake-floor sediments found in several locations, and river alluvial sediments in narrow valleys along the major stream. Volcanic sediments consist of tuffaceous sandstones, shale, breccia, and agglomerates. Lake-floor sediments comprise tuff clay, sandstones, gravel, and conglomerates. Alluvium is composed of clay, silt, sandstone, and gravel, generally originating from tertiary sediments and materials from ancient volcanic eruptions. (3) The minimum temperature in the middle stream areas varies from 15.3°C to 27°C, with an average annual rainfall ranging between 1,000 mm and 4,000 mm.

In the downstream section, (1) the topography of the lower segment is characterized by plains, undulating hills, and steep slope hills at various elevations ranging from 200 to 1,200 meters above sea level. All tributaries of the Citarum River follow a south-to-north direction, originating in Mount Burangrang, Tunggul Hill, and Canggah, and they converge at the river mouth on the north coast of the Java Sea. (2) Geological composition in the downstream Citarum area is predominantly comprised of tertiary sediments and materials resulting from ancient volcanic eruptions. (3) The average minimum temperature in the downstream region of lowland/coastal areas is 27°C, and the average annual rainfall in the mountainous areas of the upper basin is 1,000 mm. (4) In the plains/valleys along the Citarum River, the soil is formed from river sediment material. It always gets new additions when a flood occurs, so the material is relatively new (recent). Therefore, the alluvial soil (entisol and inceptisol) will be found along the Citarum River.

### 2.2. Data Sources and Processing

A combination of software applications was used to process the data, including ArcGIS version 10.3, the InVEST-SDR model, R Studio, and Geodetector [27, 31, 35, 41]. The study used data from several institutions and online sources, including satellite imagery, published maps, statistical data, and published literature.

InVEST-SDR is a spatial tool that analyzes how changes in soil erosion can impact benefits to humans, and geographic information systems (GIS) provide the spatial distribution of these changes in soil erosion. For InVEST-SDR, the WGS84 datum was employed as the input, and the input data underwent a conversion from vector to raster format, maintaining a spatial resolution of 30 meters. The sources of the secondary data utilized in the study, as well as the remote sensing data, are the digital elevation model (DEM) sourced from the Shuttle Radar Topography Mission (SRTM) Global (USGS) with a resolution of 30 m × 30 m. Land use/land cover (LULC) data underwent supervised classification from Landsat 8, procured from USGS and Google Earth Engine. Precipitation data, in the form of an isoerosivity map, and climate indicators were acquired from the Meteorological, Climatological, and Geophysical Agency. Soil metrics were derived from the Indonesian Soil Research Institute, while human activity data, including income and population density, were informed by the West Java Central Statistics Agency's 2010 and 2020 datasets.

A data range of one decade is the minimum period for observing soil erosion related to soil erosivity and erodibility, which are influenced by long climate data (at least ten years) and changes in land cover and use [42].

All data underwent rigorous preprocessing to ensure accuracy, such as geometric and atmospheric correction, to remove the effects of aerosols and gases. The datasets were then resampled using appropriate interpolation techniques to match spatial resolutions, maintaining the integrity of the data. Classification schemes were standardized across various datasets to ensure comparability. Furthermore, quality control checks were performed to check the accuracy using the confusion matrix as explained in (Shi et al., 2005). A detailed list of data sources and references is listed in Table 1.

### 2.3. Research Methods

This study uses the InVEST-SDR model to estimate the spatial and temporal soil erosion changes in the Citarum watershed from 2010 to 2020. The InVEST-SDR model calculates the annual soil loss on a pixel basis using the RUSLE model developed by [21]. The Geodetector model was used to evaluate and quantify the effect and interaction of the driving factors of soil erosion.

The study was conducted in three stages. In the first stage, the soil erosion of the study area for the years 2010 and 2020 was estimated using the RUSLE model within the InVEST-SDR model. In addition, the spatial and temporal characteristics of soil erosion also were evaluated.

In the second stage, soil erosion (as the dependent variable) and physical and socioeconomic data (as the independent variables) were generated as grid data in a raster format. Then, soil erosion and variable driving factor data were extracted based on 174 districts using the extract multivalue to point tool in ArcGIS. We limit the driving factors that cause soil erosion in the study area and grouped them into four categories, i.e., (1) topographic factors, (2) climate factors, (3) vegetation factors, and (4) human activities. The analysis was carried out using the R studio platform.

In the third stage, the Geodetector was employed to investigate the drivers of soil erosion in the study area. To extract information from raster data, 1,661 sampling points of the center of villages were selected using the “extract multi-value to point” function in ArcGIS.

**Figure 2:**
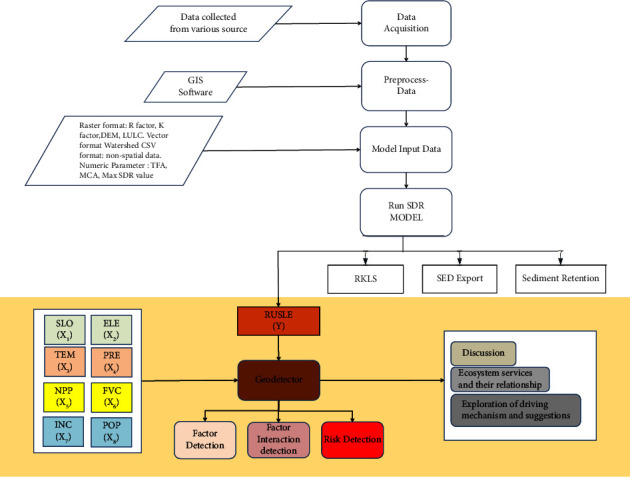
Research flowchart.

To obtain an optimal regression model, the primary task at the initial step was to select the key variables, eliminate unnecessary variables, and ensure that the explanatory variables were not multicollinearly based on the variance inflation factor (VIF) value. We excluded explanatory variables exhibiting a variance inflation factor (VIF) exceeding ten, signifying the presence of multicollinearity among the factors. Finally, the study will propose recommendations for ecological management and control measures. The flowchart indicating the research process is presented in Figure 2.

#### 2.3.1. Soil Erosion

The Citarum watershed's soil erosion modulus has been estimated using RUSLE [21]. The following is the mathematical formula:

(1) $$\begin{array}{c} A_{i}=R_{i}\times K_{i}\times {LS}_{i}\times C_{i}\times P_{i}, \end{array}$$

where *A* is defined as the average annual soil loss per unit area, expressed in tons per hectare per year (t·ha^−1^·yr^−1^), *R* is the rainfall erosivity factor, measured in mega joules millimeter per hectare per hour per year (MJ mm·ha^−1^·h^−1^·yr^−1^), *K* refers to the soil erodibility factor, with units of tons hour per mega joule per millimeter (t·h·MJ^−1^·mm^−1^), the factor LS embodies both the slope length and slope steepness, *C* is indicative of the cover and management factor, and P represents the support and conservation practice factor.

*(1) Rainfall-Runoff Erodibility Factor (R)*. The annual rainfall data from 2010 to 2019 were utilized to determine the R factor which represents the ground effect of raindrops. This was achieved using (2), as presented by [43], which involves calculating the monthly rainfall. The equation was chosen based on its suitability for the current study [20]. Also, we acknowledge the *R* factor as an indicator of the effect of rainfall on soil erosion:

(2) $$\begin{array}{c} R=\overset{12}{\underset{i=1}{\sum }}-1.15527+1.792P_{i}, \end{array}$$

where *P*_*i*_ is the monthly precipitation.

*(2) Soil Erodibility Factor (K)*. The *K* factor measures the rate of soil loss per unit of the rainfall erosivity index, with its values varying from 0 to 1. A smaller *K* value indicates a lower susceptibility to soil erosion. These *K* values were determined using (3), as formulated by Hammer in [44]:

(3) $$\begin{array}{c} K=\frac{\left\{2.713\left(M^{1.14}\right)\left(10^{-4}\right)\left(12-a\right)+3.25\;\left(b-2\right)+2.5\;\left(c-3\right)\right\}}{100}. \end{array}$$

The equation considers various factors such as soil texture, organic matter content, soil structure code, and soil permeability code. The analysis of soil texture and organic content involved assessments performed at the Soil Science Department, University of Agriculture (IPB), Indonesia. This was performed to acquire the respective values for parameters M and a. The values of parameters b and c were obtained from the soil structure and permeability codes.

*(3) Slope Length and Slope Steepness (LS)*. According to the table from Wischmeier and Smith [20], values for slope length (*L*) and slope steepness (*S*) can be obtained by calculating LS. This calculation denotes the ratio of soil loss from a specific slope, characterized by a standard length of 72.6 feet and a steepness of 9%.

*(4) Cover and Management Factor (CP)*. The relationship between soil erosion rates and agricultural methods, such as planting and tillage, is represented by a variable that spans from 1 to 0. *A* value of 1 signifies a complete lack of land cover, classifying the area as barren. On the other hand, a value nearing 0 suggests a significant protective influence, greatly reducing soil erosion. In this study, the *C* value was sourced from the *C* index table provided by Wischmeier and Smith [20] and was then integrated into the land use map.

The influence of planting and tillage practices on soil erosion rates is determined by a coefficient that ranges from 1 to 0. A coefficient of 1 indicates no land cover, identifying the area as barren land. In contrast, a coefficient approaching 0 reflects a strong protective layer, significantly safeguarding the soil from erosion. For this study, the C value was derived from the C index table by Wischmeier and Smith [20] and subsequently incorporated into the land use map.

#### 2.3.2. Geodetector Model

The Geodetector model was employed to determine the subwatersheds where soil erosion is most likely to occur, identify the key factor(s) causing soil erosion, and explore their interactions. Stepwise regression analysis is employed to examine the presence of multicollinearity among variables, selecting key variables and eliminating unnecessary ones while ensuring that the explanatory variables do not exhibit multicollinearity based on the variance inflation factor (VIF) value. The VIF is used to assess collinearity between variables: when 0 < VIF < 10, there is no multicollinearity; when 10 ≤ VIF < 100, there is strong multicollinearity; and when VIF ≥ 100, there is severe multicollinearity [45, 46].

*(1) Factor Detector*. The fundamental reasons for soil erosion (SE) in this study are categorized into four categories: topographic factors, including slope (SLO, *X*_A2_) and elevation (ELE, *X*_A2_); climate factors, such as the yearly mean temperature (TEM, *X*_A3_) and yearly mean precipitation (PRE, *X*_A4_); vegetative factors, including net primary production (NPP, *X*_A5_) and fractional vegetation cover (FVC, *X*_A6_); and human activities, such as income per capita (INC, *X*_A6_) and population density (POP, *X*_A6_). Four impact factors that have a direct impact on soil erosion were chosen for this investigation's input parameters. All variables were discretized into stratum data and other continuous variables into five categories (refer to Table S1).

In this study, the Geodetector model, recommended by [35], is employed to discern spatially varied heterogeneity of geographical strata and illustrate potential factors that could influence them. The Geodetector tool offers four different detection functions: factor, interaction, risk, and ecological detection. Information on the Geodetector model was sourced from https://www.geodetector.cn, accessed on 24 March 2023.

Our research primarily centers on utilizing the Geodetector model for interaction and factor detection. In this research, elements and interplay detectors are employed to address two key questions: identifying the significant drivers influencing soil erosion and examining the interactions among these drivers. The basic assumption of this approach is that the spatial distribution of the independent and dependent variables is nearly the same if the driving factor can significantly explain both. The Geodetector tool comprises four distinct components, i.e., (1) factor analysis, (2) interaction assessment, (3) risk evaluation, and (4) ecological detection.

The study area is characterized by spatial-stratified heterogeneity if the sum of the variance of subareas is less than that of the regional total variance. If the spatial distribution of the two variables tends to be consistent, there is a statistical correlation between them. Q-statistic in Geodetector has already been applied in many fields of natural and social sciences, which can be used to measure spatial-stratified heterogeneity, detect explanatory factors, and analyze the interactive relationship between variables [35]. The factor detector uses the *q* statistic to determine how explanatory variables may explain the dependent variable [47]. To calculate *q* values, the following formula is used:

(4) $$\begin{array}{c} q=1-\frac{\sum_{h=1}^{L}N_{h}\sigma_{h}^{2}}{{N\sigma }^{2}}, \end{array}$$

where *h* is the variable's categorization or stratification, *N*_*h*_ and *σ*^2^ are the number of samples and the variance of layer *h*, respectively, and *N* and *σ*^2^ are the variances. The symbol *q* represents the explanatory capacity of the driving factor (comprising geomorphological, meteorological, vegetation variables, and human) on the dependent variable (RUSLE), with values ranging between 0 and 1.

*(2) Interaction Detector*. The objective of the interaction detector is to ascertain whether the combined actions of two elements will increase, decrease, or maintain the existing level of influence [48]. The term “interaction detection” refers to figuring out whether *Xi* and *Xj*'s combined effects are additive, multiplicative, or neutral. The specific comparison and associated interaction relationships are shown in Table 2.

The underlying factors of SE include four main factors, i.e., (1) geomorphological factors, (2) climatic factors, (3) vegetation factors, and (4) anthropogenic factors [49, 50]. All variables' multicollinearity was evaluated using R Studio. Driving factors with VIF greater than ten were removed to lessen the impact of potential multicollinearity among variables.

*(3) Risk Detection*. Based on stratified cation differences of variables, the Geodetector model can pinpoint the areas where soil erosion is most likely to occur. The risk detector is employed to identify potential high-risk areas, while the interaction detector shows how two impact factors interact complexly [35].

The risk detector assesses if there is a notable distinction between the average values of Y in two subzones of a factor; the *t* statistic examines

(5) $$\begin{array}{c} t=\frac{{\bar{Y}}_{i=1}-\;{\bar{Y}}_{i=2}}{{\left[\text{ Var}\left({\bar{Y}}_{i=1}\right)/n_{i=1}+\text{ar}\left({\bar{Y}}_{i=1}\right)/n_{i=2}\right]}^{1/2}}. \end{array}$$

This determination is made by comparing the mean values (*Y*_*i*_) of attributes in each subregion, taking into account the number of samples (*n*_*i*_) in each subregion and the variance (Var). The *t* value, calculated using Student's *t*-test, helps determine the statistical significance of the impact of natural or anthropogenic factors at a specified significance level.

Incorporating the Geodetector model requires the segregation of continuous variables, as the model necessitates the stratification of input variables (refer to Figure 1). The model requires the inclusion of dependent and independent variables at designated sites, such as the village center. In this investigation, a total of 1,661 sampling points were employed as operational data for the implementation of the Geodetector model within ArcGIS. The Geodetector model was executed using a soil erosion map for the year 2020.

## 3. Results

### 3.1. Spatial and Temporal Transformations of Soil Erosion from 2010 to 2020

**Figure 3:**
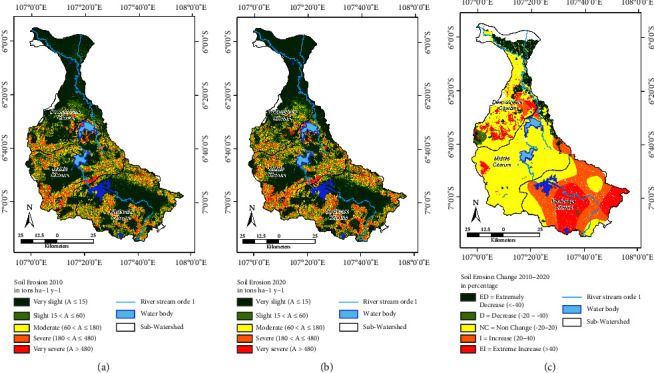
The spatial distribution of soil erosion at the grid scale: (a) in the year 2010; (b) in the year 2020; (c) change in the year 2010–2020.

In the Citarum watershed, the spatial and temporal variations in soil erosion between 2010 and 2020 are illustrated in Figure 3 and Table 3. Changes in soil erosion within the watersheds/subwatersheds of WS indicate the following in the 2010–2020 period: (a) the classification remains in the “not change” category, apart from the subwatershed scales, in which the downstream Citarum falls into the “declining” class (D). (b) Meanwhile, at the subwatershed scale, the upstream Citarum falls into the “not changed” (NC) class, the middle Citarum is classified as “decreased” (D), and the downstream Citarum is placed in the “extreme change” (EC) class.

The Citarum subwatershed had an average soil erosion of around 47.50–127.90 tons·ha^−1^·yr^−1^ in 2010, while the average erosion of the entire Citarum watershed was 93.10 tons·ha^−1^·yr^−1^ (considered moderate). Similar conditions were observed in 2020, with the Citarum subwatershed experiencing an average soil erosion of around 48.40–138.80 tons·ha^−1^·yr^−1^. The average erosion of the entire Citarum watershed was 108,60 tons·ha^−1^·yr^−1^ (considered moderate).

The Citarum watershed has shown increased soil erosion from 2010 to 2020. The Citarum watershed's total soil erosion in 2010 was 64.32 × 10^6^ tons, but by 2020, it had climbed to 75.03 × 10^6^ tons. In 10 years, there was an increase in soil erosion of 15.50 × 10^6^ tons (16.65%).

Referring to Figure 3(c), some areas (56%) are included in the change class and not changed class in the geographical distribution of soil erosion in the Citarum subwatershed between 2010 and 2020. This condition is found in almost all middle stream and downstream CW, followed by an increase in erosion of 35%, consisting of an increase of 18.71% and an extreme increase of 16.16%. This increase in the erosion class occurs in most of the upstream CW. Areas experiencing grade reduction occurred in middle CW and downstream CW areas that experienced a decrease in erosion of 9%, consisting of a decrease of 1.01% and an extreme decrease of 7.64%.

**Figure 4:**
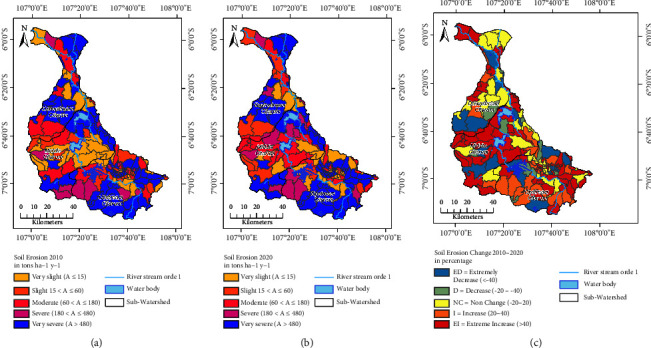
County-level soil erosion patterns: (a) distribution in 2010; (b) distribution in 2020; (c) percentage change from 2010 to 2020.

To find out more detailed erosion classes, zonal statistics are carried out based on the area of the watershed and the district area (Figure 4 and Table S2). Meanwhile, the change in soil erosion from 2010 to 2020 is shown in Table 4.

The erosion rate can be depicted in Figure 4, as indicated in Figures 4(a) and 4(b), depending on the district's size. Based on the temporal and geographical distribution trends of erosion classes between 2010 and 2020, the southern portion of the upstream watershed and the northern as well as southeastern portions of the downstream watershed exhibit a very severe erosion class. The southern portion of the upstream watershed, as well as the northern and southeastern portions of the downstream watershed, exhibits a very severe erosion class.

The very slight erosion class is relatively stable, namely, in 2010 and 2020, around 23%. The slight and moderate classes experienced a decrease compared to 2010. For the slight class, there was a decrease of 9%, while for the moderate class, the decrease reached around 20%. On the other hand, there was an increase in the severe and very severe classes. The severe class increased by 16%, while the very severe class increased by 15%.

Figure 4(c) and Table 4 show the changes in soil erosion, i.e., an increase of 56%, including a 16.09% increase and an extreme increase of 40.23%. In addition, there was a decrease of 17.24% and an extreme decrease of 7.47%. Approximately 18.97% of the area did not experience changes in soil erosion. Comparing the pixel data (0.9 ha) with the average area of the subdistrict, the rate of increase and decrease in erosion is higher. The follow-up area experienced a change of 20%, which is smaller than the pixel data (50%).

Referring to [51] in assessing the validity of the RUSLE model, the paper compared the average erosion to previously published data. In 2020, the mean erosion in the research area ranged from 48.40 to 138.80 tons·ha^−1^·yr^−1^, with an average of 108.60 tons·ha^−1^·yr^−1^ (as shown in Table 3), which falls within the acceptable limits. Comparatively, other studies conducted at the same location reported average erosion rates of 122.76 tons·ha^−1^yr^−1^ and 102.00 tons·ha^−1^·yr^−1^ in the flow area [40, 52]. These results indicate the reliability of the RUSLE model in the research area.

### 3.2. Analysis of Soil Erosion Heterogeneity

According to the results of stepwise regression analysis, a multicollinearity test was performed among the explanatory variables, as shown in Table S3. Based on Table S3, of 8 variables tested for multicollinearity, a VIF value of 1–4 was obtained (less than 10), indicating no multicollinearity [45, 46].

Meanwhile, the factor detector can be used to rank the relevance of the following factors in terms of their explanatory capacity for the geographical differentiation of soil erosion, as shown in Table S4. The statistical value is expressed as the q value. The higher the *q* value, the stronger the explanatory power of the analyzed variable.

According to Table S4, the influencing factors were ranked in the following order: vegetative factors, climatic conditions, topographic considerations, and human activities. All of these factors were found to be significant (*p* < 0.01). Vegetative factors had the greatest effect, contributing 60–89% to soil erosion. On the other hand, human activities had the least influence compared to the other factors, contributing only 11–22% each. Between these two factors, the income level (income per capita) in soil erosion had a greater influence than in population density (POP).

When circumstances in each subwatershed are examined, the same pattern emerges: vegetation has the greatest influence, followed by climate, geography, and human activity. More precisely, as shown in Table S4, overall results based on factor identification suggest the following factors.

In upstream CW, in descending order of the *q* value, the main ones causing soil erosion are FVC (75.56%) > PRE (69.06) > TEM (62.43%) > NPP (62.15%) > INC (22.77%) > SLO (22.30%) > ELE (21.28%) > POP (20.22%). In middle stream CW, in descending order of the *q* value, the main ones causing soil erosion are NPP (65.46%) > FVC (61.86%) > PRE (54.29%) > TEM (53.09%) > SLO (39.30%) > ELE (33.60%) > INC (15.75%) > POP (5.88%), whereas in downstream CW, the dominant factors are PRE (82.42%) > NPP (81.30%) > FVC (80.40%) > TEMP (46.32%) > SLO (38.37%) > POP (33.83%) > ELE (31.03%) > INC (20,28%).

### 3.3. Analysis of Soil Erosion Factors's Interaction Effect

Interaction detectors were used to determine how different variables interacted to affect the spatial differences in soil erosion among the eight selected components identified as potential contributing factors (see Appendix Table S5). Based on Appendix Table S5, of the 28 two factor interaction pairs, we present six interaction pairs with the highest degree of effect, as shown in Table 5.

The findings illustrate that the majority of variables interact with each other, resulting in a more significant effect on soil erosion. The output from the factor interaction detection indicate two primary forms of interactions: nonlinear enhancement (indicated by ^#^) and two-factor enhancement (indicated by ^*∗*^). The number of two-factor enhancement pairs ranges from 9 to 21 (32.14%–75.00%), while nonlinear enhancement pairs in the Citarum watershed range from 7 to 19 (25%–67.85%). In geospatial data analysis, the linear enhancement showed a linear function to every dynamic change of pixel in the images of factors. On the other hand, bivariate enhancement considers the relationship between two variables and can result in varying adjustments across the image, making it nonlinear. It showed more complex interactions for every factor in nature.

The *q* values of the interactions between the vegetation factor (FVC and NPP) and other factors are higher than the remaining factors when they act independently. The main factor driving on the spatial differentiation of soil erosion is the vegetation factor (FVC) combined with other factors in the upper CW and middle CW. In the downstream region, the interaction of PRE with others is quite significant.

Our study identified significant interactions among various factors, exerting a substantial impact on soil erosion. These interactions can be categorized into two main types: nonlinear enhancement and two-factor enhancement. The main factors were the vegetation factor, i.e., (FVC, *X*_A7_) and (NPP, *X*_A6_), combined with other factors.

In the upstream CW region, the most dominant order of factor interaction is as follows.

Nonlinear enhancement:

1. *X*_A3_ ∩ *X*_A7_ (temperature ∩ income per capita) (86.85%)
2. *X*_A3_ ∩ *X*_A8_ (temperature ∩ population density) (85.44%)

Nonlinear enhancement was dominant in the relationship between temperature and income per capita.

Two-factor enhancement:

1. *X*_A6_ ∩ *X*_A8_ (fractional vegetation cover ∩ population density) (84.05%)
2. *X*_A6_ ∩ *X*_A7_ (fractional vegetation cover ∩ income per capita) (82.91%)
3. *X*_A6_ ∩ *X*_A5_ (fractional vegetation cover ∩ net primary production) (82.79%)
4. *X*_A5_ ∩ *X*_A8_ (net primary production ∩ population density) (78.52%)

In the upstream subwatershed, two factors are included in the nonlinear enhancement group: temperature and other factors. Meanwhile, the relationship between vegetation factors (FVC and NPP) and other factors is included in the two-factor enhancement relationship.

While in the middle CW region, the sequence of the most dominant factor interactions are:

1. *X*_A4_ ∩ *X*_A7_ (precipitation ∩ income per capita) (89.93%)
2. *X*_A5_ ∩ *X*_A7_ (net primary production ∩ income per capita) (88.66%)
3. *X*_A2_ ∩ *X*_A3_ (digital elevation model ∩ temperature) (86.32%)
4. *X*_A6_ ∩ *X*_A8_ (fractional vegetation cover ∩ population density) (83.67%)
5. *X*_A6_ ∩ *X*_A7_ (fractional vegetation cover ∩ income per capita) (77.80%)
6. *X*_A6_ ∩ *X*_A3_ (fractional vegetation cover ∩ temperature) (76.40%)

The middle CW shows that the vegetation factor (FVC, NPP) has a significant influence, while the second dominant factor is the climate factor (TEM, PRE). The combination of these two factors will have a significant influence on soil erosion. However, the relationship between the two factors is included in nonlinear enhancement (does not strengthen each other), except for the factor *X*_A2_ ∩ *X*_A3_ which is included in two-factor enhancement. In the middle CW region, the relationship between precipitation and income per capita showed nonlinear enhancement, indicating a nonlinear connection between these factors that affects soil erosion.

Furthermore, different conditions occur in the downstream CW region, and the climate factor (PRE, *X*_A4_) has a dominant and significant interaction:

1. *X*_A6_ ∩ *X*_A7_ (fractional vegetation cover ∩ income per capita) (96.56%)
2. *X*_A5_ ∩ *X*_A8_ (net primary production ∩ population density) (89.11%)
3. *X*_A4_ ∩ *X*_A7_ (precipitation ∩ income per capita) (0.88.11%)
4. *X*_A4_ ∩ *X*_A6_ (precipitation ∩ fractional vegetation cover) (87.61%)
5. *X*_A4_ ∩ *X*_A5_ (precipitation ∩ net primary production) (86.43%)
6. *X*_A4_ ∩ *X*_A8_ (precipitation ∩ population density) (85.11%)

All two-factor relationships were found to be two-factor enhancement (bivariate enhancement). In the downstream CW, the dominant factors are vegetation (FVC, NPP) and precipitation (PRE). Precipitation was the dominant factor in the interaction, significantly influencing soil erosion.

Geodetector model selection overlooks the research area's particular characteristics and spatial extent, leading to limited consideration of terrain-related or finely distributed factors. This situation aligns with prior studies [51, 53] and implies that Geodetector models are better suited for spatial scales ranging from medium to national, where distinct spatial variations in geographic phenomena are more prominent.

The necessity of downscaling high-resolution data to align with coarser datasets for Geodetector analysis can obscure fine-scale spatial patterns, thereby compromising the method's ability to detect environmental heterogeneity. Consequently, this leads to a potential underestimation of the influence of localized factors, affecting the reliability of the spatial-stratified heterogeneity assessment [54]. Future studies can mitigate this limitation by incorporating advanced downscaling techniques that preserve local variation or by utilizing higher resolution datasets as they become available to enhance Geodetector's capacity to discriminate subtle spatial differences.

Our study goes beyond mere correlations and identifies the causal relationships between various factors and soil erosion. These findings provide valuable insights into the physical mechanisms underlying soil erosion, with different regions exhibiting distinct patterns of causality. It is important to note that Geodetector models may have limitations in capturing finer-scale terrain-related factors, and their suitability is more prominent at medium to national spatial scales.

### 3.4. Identifying Areas at High Risk for Soil Erosion

It is possible to identify areas with a high risk of soil erosion using a risk detector. Following the risk detection principle, the stratification of influencing factors is conducted based on the high risk of soil erosion, defined as an average soil erosion of ≥180 tons·ha^−1^·year^−1^ (Table 6).

In Table 6, it is evident that when considering the interplay of these two variables, factors such as precipitation: strata 4 (high), fractional vegetation cover: strata 1 (very low), slope: strata 4 (high), and net primary production: strata 2 (low) are present in the upper CW region. However, in the middle CW region, the predominant factor is the area with a substantial to high risk of erosion (erosion ≥180 tons/ha/years), which is primarily influenced by slope: strata 5 (very high) and precipitation: strata 4 (high). On the other hand, in the downstream area, the key contributors to the high erosion risk are slope: strata 5 (very high) and elevation: strata 4 (high).

## 4. Discussion

### 4.1. Spatial and Temporal Transformations of Soil Erosion from 2010 to 2020

Based on a comparison with previous studies, the RUSLE model's average erosion is proven to be accurate. The average annual soil erosion between 2010 and 2020 in the research area ranged from 47.50 to 127.90 tons·ha^−1^ and 48.40 to 138.80 ha^−1^, respectively (refer to Table 3).

The results align with Chaidar's research from 1990 to 2013, which reported similar average soil erosion in 2010, ranging from 62.04 to 137.66 tons·ha^−1^·yr^−1^ [55]. In addition, the erosion rate for 2020 corresponds to Khairunnisa's study, which documented an average annual soil erosion rate of 122.76 tons·ha^−1^·yr^−1^ with a range of 61 to 180 tons·ha^−1^·yr^−1^ [40]. Meanwhile, Suryanta's research shows an average annual soil erosion rate of 122.76 tons·ha^−1^·yr^−1^ [52]. These results are supported by Ambarwulan's research, which identified an annual erosion rate of 168.68 tons·ha^−1^·yr^−1^ for the land area, ranging from 8 to 740 tons·ha^−1^·yr^−1^ [56]. Consequently, the output of the RUSLE model in the research area is considered reliable.

Table 3 shows soil erosion changes in the Citarum watershed area (upstream, middle stream, and downstream) are relatively consistent. There is an increase in the very severe class by 1.5–3% (upstream and middle stream) and by 13% (downstream). The very slight class shows a significant increase of 134% (upstream) and 23% (downstream). On the other hand, there is a notable decrease in the severe class, i.e., 26.5% (middle) and 50–60% (upstream and downstream). In general, the class of soil erosion has decreased within the Citarum watershed. The vegetation element has the greatest influence and contributes to erosion reduction. Higher FVC (forest) areas correspond to a decrease in erosion, as observed in the upstream and middle-stream regions. These results align with studies by [11, 12], highlighting the significant influence of slope and vegetation characteristics on soil erosion.

According to [57, 58], changes in land use can affect soil loss. Moreover, further studies by [59] reveal that the diversity of plant species in an area affects the degree of erosion. For example, in the upper Citarum region (located in the Mandalahaji village of the Bandung subdistrict in West Java province) in 2020, the soil erosion rate was recorded as 14.95 tons·ha^−1^·yr^−1^ in areas with monoculture, while areas with agroforestry crops experienced only 1.5 tons·ha^−1^·yr^−1^ of soil loss.

According to [60], the nonchange (NC) status extends from the north to the south of the Citarum subwatershed, particularly in the central region. This is based on the class of change in soil erosion over ten years (refer to Figures 3(c) and 4(c)), covering approximately 57% of the Citarum watershed area. In addition, 8% of the Citarum watershed is categorized as extremely decreased (ED), mostly in the downstream CW zone. In contrast, 19% of the upstream portion of the Citarum subwatershed region experienced a decreased (D) status, while the whole Citarum watershed had an extreme decreased (ED) status.

The regions experiencing increased or decreased erosion are located in the lower areas compared to the subdistrict area approach, as shown in Figure 4(c), based on a pixel size of 30 m × 30 m (equivalent to 0.9 ha). Conversely, areas that do not experience changes are predominantly found in higher areas. In contrast, the areas experiencing increased erosion are concentrated in the middle and upstream regions of the Citarum watershed.

Based on the erosion classification in each subdistrict (as depicted in Figure 4(c)), erosion is observed throughout the region, affecting various subdistricts. The magnitude of class changes on the administrative scale is almost twice as large as the approach using the grid scale. These results indicate that to manage and mitigate soil erosion effectively, the administrative approach is more appropriate. This condition is closely related to human activities.

### 4.2. Analysis of Soil Erosion Heterogeneity

The Geodetector model effectively illustrated the detailed interplay among various factors influencing soil erosion. It identified key drivers contributing to the spatial variability of soil erosion in the Citarum watershed. The study revealed that factors like fractional vegetation cover (FVC), net primary productivity (NPP), temperature (TEM), and precipitation (PRE) were significant influencers of soil erosion in this region. This information is crucial for developing targeted ecological management approaches.

Based on Table S2, the highest level of explanatory power is dominated by the fractional vegetation cover factor in all subwatersheds. Generally, the vegetation factor is the dominant factor, followed by the climate factor. The primary causes of soil erosion throughout the entire Citarum watershed, which exhibits varied conditions, are vegetation and topography-related factors. Significant topographical heterogeneity exists, with the upstream section characterized by mountainous terrain with elevations exceeding 2000 m, while the central region features an undulating landscape.

As the main factor driving, the spatial variation in soil erosion is influenced by the presence of vegetation (fractional vegetation cover) combined with other factors in the upper CW and middle CW. This condition follows the research in Serayu watershed, Banjarnegara, and Wonosobo regencies, Central Java Province, which found that land use factors and conservation practices (CP) emerged as the primary determinants influencing soil erosion. As a result, it is crucial to develop a comprehensive approach to land conservation, particularly in regions with elevated soil loss rates. Vegetative measures, such as implementing agroforestry practices, utilizing cover crops, and employing grass strips, can be effective strategies. In addition, mechanical interventions, such as constructing bund terraces and bench terraces, can significantly mitigate soil erosion. By putting these strategies into action, it is feasible to tackle the issue of soil erosion and promote sustainable land management practices [61].

According to a spatial analysis study conducted by Natarajan [62] at Pettimudi Hills, Kerala, India, soil erosion is more prevalent in regions where there have been alterations in land use and land cover (LULC), as well as changes in agricultural practices. The study identified several factors contributing to soil erosion in the area, including the LS factor (slope length and steepness), the *C* factor (cover and management), and the *K* factor (soil erodibility). The expansion of tea plantations on areas with lower slopes, modifications in cropping patterns, and deforestation leading to the loosening of topsoil were identified as additional factors exacerbating the issue of soil erosion [62].

Research on Wulandari in the Cipeles and Cilutung watersheds at Majalengka Regency, West Java, shows that from 1990 to 2016, there was a decrease in forest and shrub areas by up to 5% every 10 years (turning into rice fields, settlements, nonirrigated rice fields, and plantations). These conditions cause erosion and high sediment yields [63]. It is similar to the study conducted by Ramadan and Supriatna in the upper Ci Catih Catchment, Sukabumi Regency, West Java. Escalation of erosion rates in each subbasin can be mainly attributed to decreased vegetation cover in steep slopes. In addition, the absence of conservation practices in land management further contributes to the increased erosion rates [64].

In the downstream region, the interaction of precipitation with other factors is quite significant. The combination of factors, including steep slopes, heavy precipitation, and extensive land use activities such as agriculture and deforestation, has contributed to the escalation of soil erosion rates [65]. Consequently, there has been a loss of valuable topsoil, decreased crop yields, and heightened sediment accumulation in rivers and reservoirs, adversely impacting agricultural productivity and water availability for local communities [66].

The research results at the Qinghai-Tibet Plateau, China, show that soil conservation services are crucial in preventing soil erosion and ensuring the ecological stability of a region. Among the factors affecting the distribution of these services, precipitation has the most significant impact, followed by slope, while the influence of a landform type is relatively minimal. The interaction between the annual precipitation and slope exerts the greatest influence [67].

### 4.3. Analysis of Soil Erosion Factors's Interaction Effect

The combined influence of these two factors exceeds the effect of any single factor alone, and the main interaction differs across different subwatersheds, as shown in the interaction detection results presented in Table 5. The results of the interaction detector show that nonlinear enhancement predominantly occurs in the interactions of the components that have the largest influence on interactions between two factors or bivariate enhancement, except for the middle subbasin. In the upstream region, the fractional vegetation cover factor predominates and reinforces each other when interacting with income per capita and population density. The increase in population and the level of welfare of people who depend on natural resources will increase awareness of protecting natural resources. Thus, more areas with a significant percentage of forest cover will exist, which will help prevent soil erosion.

Nevertheless, the consistent rise in human activity values in the downstream region signifies a growing influence of urbanization on soil erosion. This trend is primarily attributed to the rise in income per capita and population density, which often signify heightened human activity during urban expansion. This heightened activity results in a substantial increase in built-up and waterproof areas, reducing evapotranspiration and rainfall penetration, consequently leading to an escalation in soil erosion. Ecologically friendly agricultural techniques must be used in the near term to slow erosion rates from a social perspective. In the future, it will also be necessary to focus on initiatives to improve the capability of human resources, manage natural resources more effectively, and make use of social capital [68].

This research revealed the primary interaction between two factors on the spatial differentiation of soil erosion has shifted away from the slope factor to other variables. It is now primarily influenced by vegetation factors such as the fractional vegetation cover and net primary production. According to a recent study by [69], the Qiantang River Basin experienced soil erosion due to the interaction between precipitation and plant cover, accounting for 7.28% and 32.69% of the erosion, respectively. More details described the variables that triggered soil erosion, accounting for 17.02% and 29.30% of the observed erosion. Chu's study pinpointed vegetation cover, land use, and slope as the key governing factors influencing soil erosion. Conversely, the most substantial interactive control factor was identified as the combination of land use, slope, and vegetation cover in the research conducted by [70].

The relationship between the vegetation cover and slope within each subbasin was found to have a significant influence on soil erosion. According to [1], the outcomes of a previous investigation indicated a significant rise in the rate of soil erosion rises as the slope increases. Moreover, diverse forms of land use exhibited considerable variations in their impact on soil erosion. His research has reported that the augmentation of plant cover through ecological engineering significantly influences soil erosion, both directly and indirectly. The study suggests that this impact accounts for nearly half of the spatial variation in soil erosion [71].

Two measures have recently been implemented in the Citarum watershed to lessen soil erosion. The first measure to prevent soil erosion on slopes was through biological measures, namely, afforestation. Second, technical solutions, including installing mud dams, have been employed to mitigate soil erosion in the canals. Reforestation and restoration initiatives that employ native vegetation contribute significantly to the reduction of soil erosion, primarily through the augmentation of plant coverage. This stands in contrast to the construction of terraces, which mitigates soil erosion on inclined agricultural terrain by minimizing slopes and retaining soil particles [72].

Soil conservation practices used by the community are strongly tied to slope conditions and changes in agricultural land [73] to conserve natural resources. Agricultural land with less than 15% slope is conserved using chemical and vegetative methods. In contrast, agricultural land with a slope of more than 40% is conserved using mechanical, vegetative, and chemical methods. Meanwhile, tree canopies may effectively intercept rainfall and increase water supplies, and vegetation can prevent soil erosion and stabilize its structure [74]. As a result, habitat quality, water supply, and soil conservation are mutually beneficial.

According to the features of the geographical distribution of soil erosion and the discovered dominating variables in this study, each of the high-risk locations for soil erosion has its own set of factors. Referring to Table 6, the three subwatershed areas have the highest erosion risk caused by the topography factor (slope), climate factor (precipitation), and vegetation factor (net primary production or fractional vegetation cover). The three main causes of soil erosion in the downstream and middle-stream sections are slope, rainfall, and net primary production. Meanwhile, in the upstream area, the factors causing soil erosion are precipitation, net primary production, and slope.

Slope and plant cover have an explanatory power of more than 55% for soil erosion, according to the results of the interaction detection. Combining slopes and plant cover will help to more effectively control soil erosion. Soil erosion is higher on steep slopes with low vegetation cover than on steep slopes with dense vegetation cover. The primary variable impacting the spatial distribution of soil erosion in the region has been discovered to be vegetation cover [75]. The canopies intercepted the rainfall, prolonging infiltration time and reducing runoff. On the other hand, roots increase the resistance of soil structure to erosion. As a result, vegetation cover is a delicate aspect that affects soil erosion.

The implementation of fractional vegetation cover (*X*_A6_) in conjunction with precipitation (*X*_A4_) has been found to effectively mitigate soil erosion in hilly topography (upstream). Areas characterized by mountains, extensive forest cover, and high precipitation can mitigate soil erosion. The result provides significant corroboration to the outcomes of the investigation conducted by Santoso, A. D., in 2019. The Ciliwung region of West Java exhibits a clear correlation and interaction between nonvegetation settlements and agricultural areas with suboptimal soil quality, high precipitation levels, and a heightened susceptibility to substantial erosion. Despite the region's flat topography, the soil erodibility level is considerably high, leading to its susceptibility to erosion, as noted by [76]. Rudianto's research in the Dieng Plateau region of Central Java, Indonesia, has also demonstrated this. Converting forest land into agricultural or shrubland areas is associated with an increased risk of soil loss, as evidenced by the analysis of soil loss estimates [77]. A study by [78] found that vegetation had the most significant influence on soil erosion. This suggests that it is imperative to prioritize protecting and managing vegetation to safeguard soil and water.

Understanding how factors interact to enhance the effect of single factors is essential. This knowledge can inform more effective land management practices. For example, in the upper and middle Citarum watersheds, combining vegetation management (FVC) with other factors may reduce soil erosion significantly. In the downstream region, the interaction of climate (PRE) with other factors is crucial, indicating the importance of climate-sensitive erosion control measures [79, 80].

Recognizing the differential impact of controlling factors on soil erosion across subwatersheds is crucial. Tailored erosion control strategies should be developed for each subwatershed, focusing on the factors with the greatest influence in those areas. For instance, upstream and middle subwatersheds may require more emphasis on vegetation and climate management, while downstream areas should prioritize climate-related interventions [79–81].

### 4.4. Effect of Identifying Areas at High Risk for Soil Erosion

The description of the upstream subwatershed can be augmented by an additional 50% of its spatial distribution by incorporating soil erosion in conjunction with fractional vegetation cover, slopes, and rainfall. The authors of [82] found that soil erosion is primarily influenced by vegetation and topographical conditions. The rate of soil erosion depends on the heterogeneity of both conditions [82]. Gao's research has proven that steep slopes are more susceptible to soil erosion, while areas with substantial vegetation coverage effectively mitigate soil erosion. The recognition of soil erosion was significantly enhanced by integrating vegetation coverage and slope, as reported by [83].

Identifying the dominant factors influencing erosion risk in different areas allows for more precise risk assessments and targeted interventions. Engineering solutions like terracing and sediment retention structures may be effective in areas where slope, elevation, and precipitation are the main drivers. Managing rainfall and fractional vegetation cover (FVC) is essential in upstream areas. These insights can guide resource allocation for erosion risk reduction efforts [79].

The increase in soil erosion over the decade highlights the need for targeted erosion mitigation strategies. Authorities and stakeholders should prioritize investments in erosion control measures, such as reforestation and terracing, to curb the escalating erosion rates. This may involve implementing stricter land use regulations and sustainable land management practices [80, 84]. These results provide valuable information for policymakers, land managers, and conservationists to develop region-specific strategies for soil erosion control and sustainable land management practices in the Citarum watershed.

### 4.5. The Suggestions Gained from This Research

Land use planning can be customized for subwatersheds based on the identified controlling factors. For example, when fractional vegetation cover (FVC) is crucial, land use regulations can prioritize reforestation and afforestation projects. In regions with dominant climate factors (precipitation), planning can focus on water management and flood control measures [85]. The research can help define erosion risk zones, enabling land planners to restrict high-risk activities like construction or deforestation in vulnerable areas while encouraging more sustainable land use practices in low-risk zones [86].

Conservation efforts can be concentrated on addressing the factors contributing most to soil erosion. This may involve the restoration of natural vegetation, improving soil and water conservation practices, and implementing erosion control measures such as terracing and sediment basins [80, 86]. Conservation initiatives can prioritize areas with the highest erosion risk, focusing on those influenced by slope, rainfall, and vegetation cover. Conservation organizations and government agencies can target these areas with reforestation projects and erosion control interventions [87].

The research can influence policy decisions to enact and enforce regulations that protect critical factors like vegetation cover and climate stability. Stricter land use regulations, zoning laws, and incentives for sustainable land management can be implemented [87]. Meanwhile, climate change adaptation: in regions where climate factors significantly influence erosion, policymakers can integrate climate change adaptation strategies into land use and conservation policies. This might include developing early warning systems for extreme weather events or investing in climate-resilient infrastructure [88].

The results provide a data-driven foundation for informed decision-making in land use planning, conservation efforts, and policy development. By tailoring strategies to specific subwatersheds and addressing the dominant factors influencing erosion, the Citarum watershed and similar regions can work towards more sustainable and resilient land management practices.

## 5. Conclusions

In this study, we utilized the InVEST model to analyze soil erosion patterns over the period between 2000 and 2010 across various areas within the Citarum watershed. In addition, we employed the Geodetector model to assess the influence of factors such as topography, climate, vegetation, and human activities on soil erosion within the same watershed from 2010 to 2020. Our analysis provided the following findings:

1. From 2010 to 2020, soil erosion in the Citarum watershed experienced an increasing trend. In 2010, the total soil erosion was 64.32 × 10^6^ tons, while in 2020, the total soil erosion of the Citarum watershed increased to 75.03 × 10^6^ tons. Over the ten years, there was an increase in an soil erosion of 15.50 × 10^6^ tons (16.65%).
2. The magnitude of the influence of driving factors that influence soil erosion varies greatly in various subwatersheds. The vegetation factor (FVC) and climate factor (PRE) have the strongest influence on soil erosion, while human activity (POP) has the lowest explanatory power. In the upstream and middle CW, the main ones causing soil erosion are FVC, followed by NPP, PRE, TEM, SLO, ELE, POP, and INC. Meanwhile, in downstream CW, the dominant factor is PRE > NPP > FVC > TEMP > SLO > POP > ELE > INC.
3. The combined effects of various factors can amplify the impact of individual factors. The main factor driving on the spatial differentiation of soil erosion is the vegetation factor (FVC) combined with other factors in the upper CW and middle CW. Meanwhile, in the downstream region, the interaction of PRE with others is quite significant.
4. The slope, precipitation, and fractional vegetation cover primarily influence the three subwatershed areas with a high erosion risk. In the downstream and middle-stream areas, the dominant factors are caused by slope: strata 5 (very high), precipitation: strata 4 (high) (for middle CW), and elevation: strata 4 (high) (for downstream). While in the upstream area, the factors contributing to the high risk of soil erosion are precipitation: strata 4 (high), fractional vegetation cover: strata 1 (very low), slope: strata 4 (high), and net primary production: strata 2 (low).
5. These conclusions provide a more comprehensive understanding of the spatiotemporal variability of soil erosion and sediment delivery for land managers and policymakers in developing effective soil conservation strategies and enhancing the resilience of ecosystems and communities in the face of environmental changes based on various subwatershed characteristics.

## Figures and Tables

**Table 1:** List of required data inputs for the InVEST-SDR and Geodetector models.

Data description	Source	Processing	Spatial resolution
*(a) InVEST-SDR model*			
Digital elevation model	Shuttle Radar Topography Mission (SRTM) Global (USGS)	—	30 m × 30 m
Land use/land cover (LULC) data	Landsat/8 OLI imageries with acquisition date in 2000 (https://www.usgs.gov/ and Google Earth Engine)	Supervised classification	30 m × 30 m
Precipitation data	Meteorological, Climatological, and Geophysical Agency	Numeric or tabular data, along with location coordinates, are processed using a spline interpolation method	30 m × 30 m
Isoerosivity map (*R* factor)			
Soil data	Indonesian Soil Research Institute (the soil map type of 1 : 100,000)	Vector file	30 m × 30 m
Soil erodibility map (*K* factor)			
Watershed boundary data^*∗*^	Citarum-Ciliwung Watershed Management Center	Vector file	—
Data biophysical table (PUSLE and CUSLE)	FAO.org	(.csv)	30 m × 30 m

*(b) Geodetector model*			
*(1) Physical data*			
*(1.1) Topographic factors*			
Slope (variable name: *X*_A1_)	Shuttle Radar Topography Mission (SRTM) Global (USGS)	Calculation from DEM (*X*2)	30 m × 30 m
Digital elevation model (DEM) (variable name: *X*_A2_)	Shuttle Radar Topography Mission (SRTM) Global (USGS)	—	30 m × 30 m
*(1.2) Climate factor*			
Annual average temperature (TEM) (variable name: *X*_A3_)	Meteorological, Climatological, and Geophysical Agency	Numeric or tabular data, along with location coordinates, are processed using a spline interpolation method	30 m × 30 m
Annual average precipitation, PRE (variable name: *X*_A4_)	Meteorological, Climatological, and Geophysical Agency	Numeric or tabular data, along with location coordinates, are processed using a spline interpolation method	30 m × 30 m
*(1.3) Vegetation factor*			
Net primary production (NPP) (variable name: *X*_A5_)	MODIS-MOD17A3HGF V6.1 product in 2000, 2010, and 2020 (https://www.usgs.gov/ and Google Earth Engine)	The sum of all 8-day net photosynthesis is the difference between the gross primary productivity and maintenance respiration. Resampling from 500 m × 500 m	30 m × 30 m
Fractional vegetation cover (FVC) (variable name: *X*_A6_)	MODIS-MOD13Q1 with from NDVI value in 2000, 2010, and 2020 (https://www.usgs.gov/ and Google Earth Engine)	FVC = ((NDVI − 0.2)/0.3) *∗* 100. Resampling from 250 m × 250 m	30 m × 30 m
*(2) Human activities*			
Income per capita (variable name: *X*_A7_)	West Java Central Statistics Agency 2010 and 2020	Numerical/table data	30 m × 30 m
Population density (variable name: *X*_A8_)	West Java Central Statistics Agency 2010 and 2020	Numerical/table data	30 m × 30 m

^*∗*^To get more detailed erosion class data, the watershed boundaries with subdistricts are used.

**Table 2:** Interaction types of two driving factors.

Description	Interaction	Description
*q*(*X*_A1_ ∩ *X*_A2_) *<* min(*q*(*X*_A1_), *q*(*X*_A2_))	Nonlinear reduction	The influence of one variable is lessened due to the nonlinear interplay between two variables
Min(*q*(*X*_A1_), *q*(*X*_A2_)) *<* *q*(*X*_A1_ ∩ *X*_A2_) *<* max(*q*(*X*_A1_)), *q*(*X*_A2_)	Single-factor nonlinear reduction	When two univariate variables interact, the effect of an individual variable is reduced
*q*(*X*_A1_ ∩ *X*_A2_) *>* max(*q*(*X*_A1_), *q*(*X*_A1_))	Two-factor enhancement	Conversely, the interaction of two bivariate variables amplifies the impact of a single variable
*q*(*X*_A1_ ∩ *X*_A1_) = *q*(*X*_A1_) + *q*(*X*_A1_)	Independent	The relationship between two variables operates independently
*q*(*X*_A1_ ∩ *X*_A1_) > *q*(*X*_A1_) + *q*(*X*_A1_)	Nonlinear enhancement	A nonlinear interaction between two variables increases the effect of an individual variable

**Table 3:** Changes in soil erosion throughout the time between 2010 and 2020.

Name of subwatershed	Area (ha)	The year 2010		The year 2020		Delta 2010–2020	
		Mean (tons·ha^−1^)	Total (×10^6^ tons)	Mean (tons·ha^−1^)	Total (×10^6^ tons)	Mean (tons·ha^−1^)	Percentage (%)
Upstream CW	245,413	93.70	23.00	120.40	29.55	26.70	28.50
Middle-stream CW	251,373	127.90	32.15	138.80	34.89	10.90	8.52
Downstream CW	194,130	47.50	9.22	48.40	9.40	0.90	1.89
Citarum watershed	690,916	93.10	64.32	108.60	75.03	15.50	16.65

**Table 4:** Classes of soil erosion and changes between 2010 and 2020 (percent).

Years	Very slight	Slight	Moderate	Severe	Very severe
2010	22.80	14.09	53.97	9.14	0
2020	23.02	5.82	30.68	25.39	15.08
Change 2010–2020	Extremely decreased	Decreased	Not change	Increased	Extremely increased
Subdistricts (number)	30	13	33	28	70
Subdistricts (%)	17.24	7.47	18.97	16.09	40.23

**Table 5:** Interactive determination of dominant factors under different subwatersheds.

Dominant interaction	Upstream CW	Middle CW	Downstream CW
First	*X*_A3_ ∩ *X*_A7_ (0.8685)^#^	*X*_A4_ ∩ *X*_A7_ (0.8993)^#^	*X*_A6_ ∩ *X*_A7_ (0.9656)^*∗*^
Second	*X*_A3_ ∩ *X*_A8_ (0.8544)^#^	*X*_A5_ ∩ *X*_A7_ (0.8866)^#^	*X*_A5_ ∩ *X*_A8_ (0.8911)^*∗*^
Third	*X*_A6_ ∩ *X*_A8_ (0.8405)^*∗*^	*X*_A2_ ∩ *X*_A3_ (0.8632)^*∗*^	*X*_A4_ ∩ *X*_A7_ (0.8811)^*∗*^
Fourth	*X*_A6_ ∩ *X*_A7_ (0.8291)^*∗*^	*X*_A6_ ∩ *X*_A8_ (0.8367)^#^	*X*_A4_ ∩ *X*_A6_ (0.8761)^*∗*^
Fifth	*X*_A5_ ∩ *X*_A6_ (0.8279)^*∗*^	*X*_A6_ ∩ *X*_A7_ (0.7778)^#^	*X*_A4_ ∩ *X*_A5_ (0.8643)^*∗*^
Sixth	*X*_A5_ ∩ *X*_A8_ (0.7852)^*∗*^	*X*_A3_ ∩ *X*_A7_ (0.7640)^#^	*X*_A4_ ∩ *X*_A8_ (0.8511)^*∗*^

^*∗*^Bivariate enhancement; ^#^nonlinear enhancement. *Note.* Slope (*X*_A1_), digital elevation model (*X*_A2_), temperature (*X*_A3_), precipitation (*X*_A4_), net primary production (*X*_A5_), fractional vegetation cover (*X*_A6_), income per capita (*X*_A7_), and population density (*X*_A8_).

**Table 6:** Factors that are categorized as being at high risk in various subwatersheds and the average erosion.

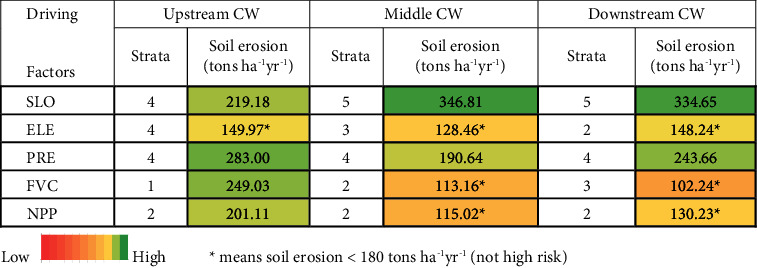

*Note.* - means soil erosion ≥180 tons·ha^−1^·yr^−1^ (high risk). Slope (*X*_A1_), digital elevation model (*X*_A2_), temperature (*X*_A3_), precipitation (*X*_A4_), net primary production (*X*_A5_), fractional vegetation cover (*X*_A6_), income per capita (*X*_A7_), and population density (*X*_A8_).

## Data Availability

The data used to support the findings of this study are within the paper.

## Supplementary Materials

Supplementary Materials Table S1: stratification of the contributing factors that cause soil erosion. Table S2A: the distribution of soil erosion intensity across different categories of watersheds in the year 2010. Table S2B: the distribution of soil erosion intensity across different categories of watersheds in the year 2020. Table S2C: the distribution of soil erosion intensity across different categories of watersheds in the years 2010 and 2020 (%). Table S3: a test for multicollinearity between the explanatory factors. Table S4: *q* value of each driving factor of soil erosion at the Citarum watershed. Table S5: interactive determination of dominant factors under different subwatersheds.
