# Supplementary material for: Analysis of Factors Influencing Spatial Distribution of Soil Erosion under Diverse Subwatershed Based on Geospatial Perspective: A Case Study at Citarum Watershed, West Java, Indonesia
Source: Scientifica (Cairo). 2024 Jan 11;2024:7251691. doi: 10.1155/2024/7251691 (PMC11221964; doi:10.1155/2024/7251691)
Supplement: Supplementary Materials — Table S1: stratification of the contributing factors that cause soil erosion. Table S2A: the distribution of soil erosion intensity across different categories of watersheds in the year 2010. Table S2B: the distribution of soil erosion intensity across different categories of watersheds in the year 2020. Table S2C: the distribution of soil erosion intensity across different categories of watersheds in the years 2010 and 2020 (%). Table S3: a test for multicollinearity between the explanatory factors. Table S4: q value of each driving factor of soil erosion at the Citarum watershed. Table S5: interactive determination of dominant factors under different subwatersheds. [file 7251691.f1.zip › Table_S1.docx]

**Table S1.** Stratification of the contributing factors that cause soil erosion.

| **Middle stream CW** | SLO  (%) | ELE  (m asl) | TEM  (^o^C) | PRE  (mm/year) |
| --- | --- | --- | --- | --- |
| Very Slight | 0-5 | < 400 | < 18 | 791-1475 |
| Slight | 5- 15 | 400-800 | 18-21 | 1.475-1.820 |
| Moderate | 15-30 | 800-100 | 22-24 | 1.820-2.250 |
| Severe | 30-45 | 1000-1500 | 24-25 | 2.250-2.750 |
| Very Severe | >45 | - 1500 | - 25 | >2.750 |
| **Middle stream CW** | NPP  (Ton/ha) | FVC  (%) | INC  (IDR million/capita) | POP (people/ha) |
| Very Slight | 0- 5,675 | >80% | 4-7.5 | 671-634 |
| Slight | 5,675-8,309 | 60-80% | 7.5-17.6 | 634-836 |
| Moderate | 8,309-11,215 | 40-60 % | 17.6-27.71 | 836-1239 |
| Severe | 11,215-14,120 | 20-40 | 27.11-37.58 | 1429-2351 |
| Very Severe | > 14,120 | 0-20 | 37.58-58. | 2351-14.916 |
