# Supplementary material for: Analysis of Factors Influencing Spatial Distribution of Soil Erosion under Diverse Subwatershed Based on Geospatial Perspective: A Case Study at Citarum Watershed, West Java, Indonesia
Source: Scientifica (Cairo). 2024 Jan 11;2024:7251691. doi: 10.1155/2024/7251691 (PMC11221964; doi:10.1155/2024/7251691)
Supplement: Supplementary Materials — Table S1: stratification of the contributing factors that cause soil erosion. Table S2A: the distribution of soil erosion intensity across different categories of watersheds in the year 2010. Table S2B: the distribution of soil erosion intensity across different categories of watersheds in the year 2020. Table S2C: the distribution of soil erosion intensity across different categories of watersheds in the years 2010 and 2020 (%). Table S3: a test for multicollinearity between the explanatory factors. Table S4: q value of each driving factor of soil erosion at the Citarum watershed. Table S5: interactive determination of dominant factors under different subwatersheds. [file 7251691.f1.zip › Table_S2A.docx]

**Tabel S2A.** The distribution of soil erosion intensity across different categories of watersheds in the year 2010.

| Erosion Intensity Level | Upstream CW | | Middle stream CW | | Downstream CW | |
| --- | --- | --- | --- | --- | --- | --- |
|  | Area (ha) | % | Area (ha) | % | Area (ha) | % |
| Very Slight | 56.710 | 23,11 | 24.124 | 9,60 | 81.660 | 42,06 |
| Slight | 52.661 | 21,46 | 37.990 | 15,11 | 77.667 | 40,01 |
| Moderate | 113.637 | 46,30 | 145.822 | 58,01 | 34.804 | 17,93 |
| Severe | 20.418 | 8,32 | 43.437 | 17,28 | 0 | 0 |
| Very Severe | 1.987 | 0,81 | - | - | 0 | 0 |
| Total | 245.413 | 100,00 | 251.373 | 100,00 | 194,130 | 100.00 |
