# Supplementary material for: Analysis of Factors Influencing Spatial Distribution of Soil Erosion under Diverse Subwatershed Based on Geospatial Perspective: A Case Study at Citarum Watershed, West Java, Indonesia
Source: Scientifica (Cairo). 2024 Jan 11;2024:7251691. doi: 10.1155/2024/7251691 (PMC11221964; doi:10.1155/2024/7251691)
Supplement: Supplementary Materials — Table S1: stratification of the contributing factors that cause soil erosion. Table S2A: the distribution of soil erosion intensity across different categories of watersheds in the year 2010. Table S2B: the distribution of soil erosion intensity across different categories of watersheds in the year 2020. Table S2C: the distribution of soil erosion intensity across different categories of watersheds in the years 2010 and 2020 (%). Table S3: a test for multicollinearity between the explanatory factors. Table S4: q value of each driving factor of soil erosion at the Citarum watershed. Table S5: interactive determination of dominant factors under different subwatersheds. [file 7251691.f1.zip › Table_S2B.docx]

**Tabel S2B.**  The distribution of soil erosion intensity across different categories of watersheds in the year 2020.

| Erosion Intensity Level | Upstream CW | | | | Middle stream CW | | Downstream CW | |
| --- | --- | --- | --- | --- | --- | --- | --- | --- |
|  | Area (ha) | | % | | Area (ha) | % | Area (ha) | % |
| Very Slight | 56.350 | | 22,96 | | 29.277 | 11,65 | 78347,66 | 40,36 |
| Slight | 11.710 | | 4,77 | | 5.171 | 2,06 | 26811,1 | 13,81 |
| Moderate | 92.564 | | 37,72 | | 93.471 | 37,18 | 10712,03 | 5,52 |
| Severe | 61.440 | | 25,04 | | 98.815 | 39,31 | 9913,414 | 5,11 |
| Very Severe | 23.350 | | 9,51 | | 24.638 | 9,80 | 68345,8 | 35,21 |
| Total | 245,413 | | 100.00 | | 251,373 | 100.00 | 194,130 | 100.00 |
|  | |  | |  |  |  |  |  |
