# Supplementary material for: Analysis of Factors Influencing Spatial Distribution of Soil Erosion under Diverse Subwatershed Based on Geospatial Perspective: A Case Study at Citarum Watershed, West Java, Indonesia
Source: Scientifica (Cairo). 2024 Jan 11;2024:7251691. doi: 10.1155/2024/7251691 (PMC11221964; doi:10.1155/2024/7251691)
Supplement: Supplementary Materials — Table S1: stratification of the contributing factors that cause soil erosion. Table S2A: the distribution of soil erosion intensity across different categories of watersheds in the year 2010. Table S2B: the distribution of soil erosion intensity across different categories of watersheds in the year 2020. Table S2C: the distribution of soil erosion intensity across different categories of watersheds in the years 2010 and 2020 (%). Table S3: a test for multicollinearity between the explanatory factors. Table S4: q value of each driving factor of soil erosion at the Citarum watershed. Table S5: interactive determination of dominant factors under different subwatersheds. [file 7251691.f1.zip › Table_S2C.docx]

**Table S2C.** The distribution of soil erosion intensity across different categories of watersheds in the year 2010 and 2020 (%)

| Erosion Intensity Level | Upstream CW | | Middle stream CW | | Downstream CW | |
| --- | --- | --- | --- | --- | --- | --- |
|  | Area (ha) | % | Area (ha) | % | Area (ha) | % |
| Very Slight | -360 | - 0,63 | 5.153 | 21,36 | -3.312 | - 4,06 |
| Slight | -40.951 | - 77,76 | -32.819 | - 86,39 | -50.856 | - 65,48 |
| Moderate | -21.073 | - 18,54 | -52.351 | - 35,90 | -24.092 | - 69,22 |
| Severe | 41.022 | 200,91 | 55.378 | 127,49 | 9.913 | 100,00 |
| Very Severe | 21.363 | 1.075,14 | 24.638 | 100,00 | 68.346 | 100,00 |
| Total | 245,413 | 100.00 | 251,373 | 100.00 | 194,130 | 100.00 |
