# Supplementary material for: Analysis of Factors Influencing Spatial Distribution of Soil Erosion under Diverse Subwatershed Based on Geospatial Perspective: A Case Study at Citarum Watershed, West Java, Indonesia
Source: Scientifica (Cairo). 2024 Jan 11;2024:7251691. doi: 10.1155/2024/7251691 (PMC11221964; doi:10.1155/2024/7251691)
Supplement: Supplementary Materials — Table S1: stratification of the contributing factors that cause soil erosion. Table S2A: the distribution of soil erosion intensity across different categories of watersheds in the year 2010. Table S2B: the distribution of soil erosion intensity across different categories of watersheds in the year 2020. Table S2C: the distribution of soil erosion intensity across different categories of watersheds in the years 2010 and 2020 (%). Table S3: a test for multicollinearity between the explanatory factors. Table S4: q value of each driving factor of soil erosion at the Citarum watershed. Table S5: interactive determination of dominant factors under different subwatersheds. [file 7251691.f1.zip › Table_S3.docx]

**Table S3.** A test for multicollinearity between the explanatory factors

|  | topographic factors | | climate factors | | vegetative factors | | human activities | |
| --- | --- | --- | --- | --- | --- | --- | --- | --- |
|  | X_A1_ | X _A2_ | X _A3_ | X _A4_ | X _A5_ | X _A6_ | X _A7_ | X _A8_ |
| Upstream CW | 1.640 | 1.434 | 1.196 | 1.536 | 1.223 | 4.969 | 3.587 | 1.982 |
| Middle stream CW | 1.986 | 2.095 | 2.232 | 3.636 | 3.615 | 2.393 | 1.860 | 1.531 |
| Downstream CW | 1.909 | 1.427 | 1.713 | 2.571 | 3.691 | 2.309 | 1.688 | 8.083 |

Note: Slope (X_A1_), Digital elevation model (X_A2_),Temperature (X_A3_), Precipitation (X X_A1_), Net Primary Production (X_A5_), Fractional Vegetarion Cover (X_A6_), Income per capita (X_A7_), Population density (X_A8_)
