# Supplementary material for: Analysis of Factors Influencing Spatial Distribution of Soil Erosion under Diverse Subwatershed Based on Geospatial Perspective: A Case Study at Citarum Watershed, West Java, Indonesia
Source: Scientifica (Cairo). 2024 Jan 11;2024:7251691. doi: 10.1155/2024/7251691 (PMC11221964; doi:10.1155/2024/7251691)
Supplement: Supplementary Materials — Table S1: stratification of the contributing factors that cause soil erosion. Table S2A: the distribution of soil erosion intensity across different categories of watersheds in the year 2010. Table S2B: the distribution of soil erosion intensity across different categories of watersheds in the year 2020. Table S2C: the distribution of soil erosion intensity across different categories of watersheds in the years 2010 and 2020 (%). Table S3: a test for multicollinearity between the explanatory factors. Table S4: q value of each driving factor of soil erosion at the Citarum watershed. Table S5: interactive determination of dominant factors under different subwatersheds. [file 7251691.f1.zip › Table_S4.docx]

**Table S4**. q value of each driving factor of soil erosion at the Citarum Watershed

|  | X_A1_ | X _A2_ | X _A3_ | X _A4_ | X _A5_ | X _A6_ | X _A7_ | X _A8_ |
| --- | --- | --- | --- | --- | --- | --- | --- | --- |
| Upstream CW | 0.223 | 0.2128 | 0.6243* | 0.6906* | 0.6215* | 0.7556* | 0.2277 | 0.2022 |
| Middle stream CW | 0.393 | 0.336 | 0.5309* | 0.5429* | 0.6546* | 0.6186* | 0.1575 | 0.0588 |
| Downstream CW | 0.3837 | 0.3103 | 0.4632* | 0.8242* | 0.8130* | 0.8040* | 0.2028 | 0.3383 |
| Average | 0.3332 | 0.2864 | 0.5395 | 0.6859 | 0.6964 | 0.7261 | 0.1960 | 0.1998 |

*Most high - all passed the significance test (p < 0.01).

Note: Slope (X_A1_), Digital elevation model (X_A2_),Temperature (X_A3_), Precipitation (X_A4_), Net Primary Production (X_A5_), Fractional Vegetarion Cover (X_A6_), Income per capita (X_A7_), Population density (X_A8_)
