# Supplementary material for: Analysis of Factors Influencing Spatial Distribution of Soil Erosion under Diverse Subwatershed Based on Geospatial Perspective: A Case Study at Citarum Watershed, West Java, Indonesia
Source: Scientifica (Cairo). 2024 Jan 11;2024:7251691. doi: 10.1155/2024/7251691 (PMC11221964; doi:10.1155/2024/7251691)
Supplement: Supplementary Materials — Table S1: stratification of the contributing factors that cause soil erosion. Table S2A: the distribution of soil erosion intensity across different categories of watersheds in the year 2010. Table S2B: the distribution of soil erosion intensity across different categories of watersheds in the year 2020. Table S2C: the distribution of soil erosion intensity across different categories of watersheds in the years 2010 and 2020 (%). Table S3: a test for multicollinearity between the explanatory factors. Table S4: q value of each driving factor of soil erosion at the Citarum watershed. Table S5: interactive determination of dominant factors under different subwatersheds. [file 7251691.f1.zip › Table_S5.docx]

**Table S5.** Interactive determination of dominant factors under different Sub Watershed.

| **Upstream CW** | X_A1_ | X_A2_ | X_A3_ | X_A4_ | X_A5_ | X_A6_ | X_A7_ | X_A8_ |
| --- | --- | --- | --- | --- | --- | --- | --- | --- |
| X_A1_ | 0.2230 |  |  |  |  |  |  |  |
| X_A2_ | 0.2619* | 0.2128 |  |  |  |  |  |  |
| X_A3_ | 0.2576 | 0.2427 | 0.6243 |  |  |  |  |  |
| X_A4_ | 0.3040* | 0.3155 | 0.7260 | 0.6906 |  |  |  |  |
| X_A5_ | 0.2680* | 0.2585* | 0.7182* | 0.7243* | 0.6215 |  |  |  |
| X_A6_ | 0.2886* | 0.2622* | 0.7201* | 0.8279* | 0.7285* | 0.7556 |  |  |
| X_A7_ | 0.2390* | 0.3356* | 0.8650 | 0.7257 | 0.7818* | 0.8291* | 0.2277 |  |
| X_A8_ | 0.2396* | 0.3380 | 0.8544 | 0.7221 | 0.7852* | 0.8405* | 0.3303* | 0.2022 |
|  |  |  |  |  |  |  |  |  |
| **Middle stream CW** | X_A1_ | X_A2_ | X_A3_ | X_A4_ | X_A5_ | X_A6_ | X_A7_ | X_A8_ |
| X_A1_ | 0.3930 |  |  |  |  |  |  |  |
| X_A2_ | 0.4258* | 0.3360 |  |  |  |  |  |  |
| X_A3_ | 0.5391 | 0.6139 | 0.5309 |  |  |  |  |  |
| X_A4_ | 0.6661 | 0.8632* | 0.6983 | 0.5429 |  |  |  |  |
| X_A5_ | 0.6128* | 0.6884* | 0.7610 | 0.7845* | 0.6546 |  |  |  |
| X_A6_ | 0.6177 | 0.6656 | 0.7640 | 0.6518* | 0.7969* | 0.6186 |  |  |
| X_A7_ | 0.6235 | 0.7389 | 0.6591 | 0.8993 | 0.8866 | 0.7778 | 0.1575 |  |
| X_A8_ | 0.4114 | 0.4905 | 0.7522 | 0.8136 | 0.7512 | 0.8367 | 0.3485 | 0.0588 |
| **Downstream CW** | X_A1_ | X_A2_ | X_A3_ | X_A4_ | X_A5_ | X_A6_ | X_A7_ | X_A8_ |
| X_A1_ | 0.3837 |  |  |  |  |  |  |  |
| X_A2_ | 0.5523 | 0.3103 |  |  |  |  |  |  |
| X_A3_ | 0.4260 | 0.4016 | 0.4632 |  |  |  |  |  |
| X_A4_ | 0.4808 | 0.4210 | 0.5345 | 0.8242 |  |  |  |  |
| X_A5_ | 0.5931 | 0.5207 | 0.5229 | 0.8643* | 0.8130 |  |  |  |
| X_A6_ | 0.5149 | 0.4231 | 0.5412 | 0.8761* | 0.8513* | 0.8040 |  |  |
| X_A7_ | 0.4285 | 0.4152 | 0.5925 | 0.8811* | 0.8812 | 0.9656* | 0.2028 |  |
| X_A8_ | 0.5278 | 0.5560 | 0.5113 | 0.8511* | 0.8911* | 0.8211* | 0.4872 | 0.3383 |

* represents bivariate enhancement; - represents non-linear enhancement.

Note: Slope (X_A1_), Digital elevation model (X_A2_),Temperature (X_A3_), Precipitation (X_A4_), Net Primary Production (X_A5_), Fractional Vegetarion Cover (X_A6_), Income per capita (X_A7_), Population density (X_A8_)
